# Supplementary material for: The effect of intrapartum prolonged oxygen exposure on fetal metabolic status: secondary analysis from a randomized controlled trial
Source: Front Endocrinol (Lausanne). 2023 Jun 27;14:1204956. doi: 10.3389/fendo.2023.1204956 (PMC10335765; doi:10.3389/fendo.2023.1204956)
Supplement: Supplementary file 3 [file DataSheet_3.zip › Result-X101SC21092977-Z01-J001-B1-42/5.MetKeggEnrichment/O.vs.A/O.vs.A_pos_KEGG_map/KEGG.map.html]

Pathway Enrichment


  

# The most enriched pathway terms of O.vs.A\_pos

Statistic method: hypergeometric test

FDR correction method: Benjamini and Hochberg

| MapID | MapTitle | Pvalue | AdjustedPv | x | y | n | N | EnrichDirect | GeneIDs |
| --- | --- | --- | --- | --- | --- | --- | --- | --- | --- |
| map00740 | Riboflavin metabolism | 0.0731707317073171 | 0.239773707819401 | 1 | 1 | 6 | 82 | Over | Com\_3007\_pos |
| map04540 | Gap junction | 0.0731707317073171 | 0.239773707819401 | 1 | 1 | 6 | 82 | Over | Com\_3997\_pos |
| map04726 | Serotonergic synapse | 0.0731707317073171 | 0.239773707819401 | 1 | 1 | 6 | 82 | Over | Com\_3997\_pos |
| map04750 | Inflammatory mediator regulation of TRP channels | 0.0731707317073171 | 0.239773707819401 | 1 | 1 | 6 | 82 | Over | Com\_3997\_pos |
| map00380 | Tryptophan metabolism | 0.0799245692731338 | 0.239773707819401 | 2 | 7 | 6 | 82 | Over | Com\_2899\_pos Com\_3997\_pos |
| map04976 | Bile secretion | 0.127793358574464 | 0.265921409214092 | 2 | 9 | 6 | 82 | Over | Com\_2445\_pos Com\_3997\_pos |
| map04721 | Synaptic vesicle cycle | 0.141824751580849 | 0.265921409214092 | 1 | 2 | 6 | 82 | Over | Com\_3997\_pos |
| map04742 | Taste transduction | 0.141824751580849 | 0.265921409214092 | 1 | 2 | 6 | 82 | Over | Com\_3997\_pos |
| map00760 | Nicotinate and nicotinamide metabolism | 0.206187895212285 | 0.281165311653116 | 1 | 3 | 6 | 82 | Over | Com\_2445\_pos |
| map00860 | Porphyrin and chlorophyll metabolism | 0.206187895212285 | 0.281165311653116 | 1 | 3 | 6 | 82 | Over | Com\_702\_pos |
| map04024 | cAMP signaling pathway | 0.206187895212285 | 0.281165311653116 | 1 | 3 | 6 | 82 | Over | Com\_3997\_pos |
| map04977 | Vitamin digestion and absorption | 0.266477422158188 | 0.333096777697735 | 1 | 4 | 6 | 82 | Over | Com\_3007\_pos |
| map04080 | Neuroactive ligand-receptor interaction | 0.322902235838327 | 0.372579502890378 | 1 | 5 | 6 | 82 | Over | Com\_3997\_pos |
| map00360 | Phenylalanine metabolism | 0.375663100578198 | 0.402496179190926 | 1 | 6 | 6 | 82 | Over | Com\_3658\_pos |
| map01100 | Metabolic pathways | 0.658128611123363 | 0.658128611123363 | 5 | 55 | 6 | 82 | Over | Com\_2445\_pos Com\_2899\_pos Com\_3007\_pos Com\_3997\_pos Com\_702\_pos |
